# Investigating the Neurological Correlates of Workplace Deviance Using a Rodent Model of Extinction

**DOI:** 10.1038/s41598-018-35748-y

**Published:** 2018-11-23

**Authors:** J. Tabor, Y. Griep, R. Collins, R. Mychasiuk

**Affiliations:** 1 University of Calgary, Faculty of Arts, Department of Psychology, Calgary, Canada; 2 Division of Epidemiology of the Stress Research Institute, Stockholm University, Stockholm, Sweden; 3 Alberta Children’s Hospital Research Institute & Hotchkiss Brain Institute, University of Calgary, Calgary, Canada

## Abstract

Employee deviance and time theft is an expensive and pervasive workplace problem. Research indicates that a primary reason employees engage in deviant behaviour is the perception of injustice often associated with psychological contract breach (i.e., broken promises). This study used a rodent model to mimic said experience of broken promises and then examined the subsequent neurophysiological changes that lead to the display of deviant behaviours. Specifically, we generated a psychological contract using a 3 choice serial reaction task, then broke the promise, and finally examined deviant behaviours and neurological correlates. After the broken promise, rats had elevated levels of corticosterone and testosterone, engaged in riskier behaviour, and were more aggressive. The most prominent changes in gene expression were associated with serotonin and stress, and were found in the nucleus accumbens. This study highlights the value of pre-clinical models in the investigation of the theoretical tenants of industrial and organizational psychology.

## Introduction

Employee deviance and time theft is an expensive and pervasive workplace problem, costing businesses between $6-200 billion per year^1^. Research indicates that one of the primary reasons employees engage in deviant behaviour is the perception of injustice or broken promises^2,3^. Unhappy workers admitted to napping on the job, watching Netflix, checking social media, shopping online, and stealing from their employer^4^. Industrial and organizational psychologists have amassed a great deal of evidence demonstrating that negative employee behaviours, such as the ones described above, are often triggered by a psychological contract breach^5,6^. Psychological contracts are stable representations of perceived promises employees believe their employers are obligated to fulfill^7^. When these promises are broken, employees attempt to ‘even the score’, and regain what is ‘owed’ to them by engaging in deviant behavior.

Psychologists have explained this reactionary behaviour using Conservation of Resources (COR) theory. The primary premise of COR theory suggests that individuals strive to obtain, retain, protect, and enhance the things (resources) that they value^8^. Within COR theory, resources are finite, and stress occurs when resources are threatened, lost, or unstable^8^. The corollary of COR theory is that those who are deprived of resources are likely to adopt defensive and aggressive behaviours to conserve their assets^8^. In other words, when organizations break their promises, employees seek retribution to regain resources they perceive to be lost^5,6,9^. This extinction stress, the stress associated with losing a reinforced reward has been studied extensively in the context of addiction (for review see^10^), but sparsely under other conditions^11^. Although the neural processes underlying both circumstances may be similar, it is important to study this phenomenon without addictive cues that can be highly motivating.

Based on COR theory, these loss related events associated with broken promises, have the potential to trigger stress. This loss of valued resources places the individual under significant stress^5^ which can trigger retaliatory behaviours^6,12,13^. It has been speculated that the increased stress, aggression, and deviant workplace behaviours that follow the experience of broken promises are mediated by changes to the serotonergic system in the brain^5,6,14^. Serotonin is involved in aggression and the establishment of social status in numerous species, including humans and rodents (for review see^15^). Given that lowered levels of serotonin are associated with increased aggression and antisocial behaviour^16,17^ as well as impaired representation of reward and punishment values^18^, the serotonergic system has significant appeal to understand the theoretical premises of COR theory and workplace deviance.

Understanding the neurological basis of deviant behaviour following the experience of broken promises is of critical importance because a substantial portion of employees experience broken promises, the outcomes of these broken promises are costly for both employees and employers, and given the subjective nature of psychological contracts, it is often difficult to prevent broken promises from occurring^19^. Although some studies have begun to examine the relationship between stress and retaliatory behaviors with respect to the experience of broken promises, these have largely been focused on self-report questionnaires^5,9,14^. Therefore, this study used a rodent model to mimic the experience of broken promises and then examined the subsequent neurophysiological changes that lead to the deviant behaviours. To do this, we generated a psychological contract—when the rat nose poked correctly it received a food pellet reward. Next, we broke this promise among half of the rats—the rat continued to nose poke correctly, but we no longer provided food pellets in return for their behavior. Finally, we examined the deviant behaviour and neurological correlates and brain changes in rats that experienced broken promises versus those that did not. We chose to examine gene expression changes in the nucleus accumbens (NAc), prefrontal cortex (PFC), and hippocampus (HPC) because these brain regions are known to be involved in the neurobiological circuits associated with reward and stress^20–22^. Moreover the PFC plays a significant role in executive decision making^23^, the HPC is involved in reinforcement and extinction behaviours^24^, and while the NAc contributes to the neurobiology of reward, it also influences aggressive behaviour^25^.

## Methods

### Subjects

All reported experiments were carried out in accordance with the Canadian Council of Animal Care and received approval from the University of Calgary Conjoint Faculties Research Ethics Approval Board. Sixteen male Sprague Dawley rats (Charles Rivers Laboratories) were caged in groups of 4 and housed in an animal husbandry room kept at 21 °C with a 12:12 hr light:dark cycle where the lights turned on at 7 AM. The animals had ad libitum access to food and water until they were calorically restricted at postnatal day 42 (P42). This diet restriction was done to provide incentive for the food reward-driven 3-Choice Serial Reaction task they were to undertake. Caloric restriction was minimal, whereby rats maintained body weights that were between 90–95% of typical developing rats at this age.

### 3-Choice Serial Reaction Testing Paradigm

At P43 all animals began training in the 3-Choice Serial Reaction test. The protocol used for this training was similar to that used by Barie *et al*.^26^ however modified for this experiment. The protocol used two identical Habitest Modular 5-Hole Operant Conditioning Chambers (28 cm × 29 cm × 24 cm–WxHxD; Harvard Apparatus, QC Canada), but only the middle 3/5 holes were utilized in this procedure. The training was divided into three stages. The first stage was designed to teach the rats that a reward (a banana flavoured 45 g precision-weight food tablet; BioServ, Product #F0059) would be delivered when they correctly nose-poked an illuminated hole. These training sessions were 15 minutes long. To begin, the rat would be placed in the operant chamber with a single pellet in the reward magazine. Once the rat retrieved the reward pellet, the session began with the house light and reward magazine light turning off, and the illuminated stimulus initiated. The stimulus in Stage 1 consisted of 1 of the 3 holes being illuminated until the rat nose-poked the specific hole. Upon a successful nose poke, the hole light would turn off, and a reward pellet was dropped into the reward magazine as the house/reward magazine lights illuminated. During Stage 1, if the rat nose-poked the wrong hole, it would not receive a reward pellet. The rat was also not penalized if it poked one of the 4 dark holes, and the illuminated stimulus would simply stay lit. When the rat nose-poked the correct hole and retrieved its reward pellet, a second hole was illuminated. Stage 1 training occurred once per day for approximately 14 consecutive days until the animals were proficient enough (consistently achieving an efficiency of approximately 70% correct nose pokes) to move on to Stage 2 training.

Stage 2 training required spatial attention as the illuminated stimulus was presented for a limited time frame. Stage 2 also began with the retrieval of a banana pellet from the reward magazine. However, in Stage 2, the illuminated stimulus was only lit for 5 seconds. The rat had to nose poke within the 5 second illumination time to receive a reward. If the rat successfully nose-poked in this time period, the hole light was extinguished, the house and reward magazine lights were illuminated and the reward pellet was dispensed. If the rat failed to nose-poke the proper hole within the 5 second time limit, it received a 3 second time-out. When the time-out ended, another illumination stimulus would light up. Stage 2 sessions also lasted for 15 minutes and occurred once per day for the next 14 consecutive days until the animals were proficient enough to move forward to Stage 3 training.

Stage 3 training further tested the rat’s proficiency of the illumination task as the stimulus was only turned on for 1 second. Stage 3 began similar to the previous two stages with the retrieval of a reward pellet and the house and reward magazine lights illuminated. This stage required the rats to be quicker between nose-pokes as the illumination time was limited to 1 second intervals. If the rat missed this illumination stimulus, it received a longer time-out of 5 seconds until the next stimulus turned on. The rats still received a reward pellet for each correct nose-poke. This stage also lasted for 15 minutes and occurred once per day for 10 consecutive days. Once proficient at this task, half of the rats were subjected to Stage 4 (Broken Promise), and the other half remained on Stage 3 (Kept Promise).

Broken Promise - Stage 4. Stage 4 was an Extinction protocol that occurred over 3 consecutive days which was implemented onto half of the rats in the Broken Promise group. This stage was designed to determine if the rats would continue to work and nose-poke in the absence of a reward. The rats were placed in the chambers for 15 minute sessions. The Extinction protocol was identical to Stage 3. However, when the rat correctly nose-poked in the 1 second interval, the light would extinguish followed by illumination of the house and reward magazine lights, but a reward pellet was NOT provided. Following this, another stimuli would illuminate in a different hole. If the rat missed the correct hole, there was also a 5 second time-out between Go stimuli, similar to Stage 3. Rats were randomly placed into each group, with each cage having 2 animals in the Broken Promise group and 2 in the Kept Promise group. A total of 16 rats were used in this study, (Promise Broken *n* = 8; Kept Promise *n* = 8)

All data for the proficiency aspect of Stage 3 and Extinction protocol was collected and analyzed with Graphic State 4 software (Coulburn Instruments, QC Canada). Rats were scored on their levels of efficiency (the number of correct nose-pokes over the total number of stimuli presented in the testing session) in the 5 days leading up to the Extinction protocol, as well as the 3 days after the Extinction protocol started. These efficiencies were averaged over all the animals in both the Broken Promise group and the control group (Kept Promise) in both pre- and post-extinction phases. The total number of nose-pokes, duration of time spent in ‘time-out’, and the number of trials completed for each rat was also collected.

### Dominance Tube

Aggression levels were measured using the dominance tube test, which was administered immediately after each of the Stage 4 testing sessions (P77–79). Rats were released into opposite ends of a clear tube, narrow enough that the animals didn’t have room to turn around. The rats met in the middle of the tube, and the more dominant animal exhibited more aggression by pushing forward to force their opponent out of the tube. An animal was declared the loser when all four of its paws were out of the tube and the winner was the one remaining inside. There was a total of 3 trials per match up and the rats faced a new cage-mate each day so that by the end of the 3 days they have faced-off against all animals in their cage. There were no significant differences in rat weight between cage-mates. Trial wins, win percentage, and time spent in the tube was recorded for each animal.

### Open Field

Rats were tested in the Open Field paradigm on P79, prior to testing in the dominance tube, to measure general locomotor activity. Animals were placed in the center of a circular arena with a diameter of 135 cm, and were permitted to explore the environment for 10 minutes. An over-head camera equipped with Noldus Ethovision XT 10.0 software was used to track and analyze the rat’s overall movement and distance travelled. Virkon® was used to clean the arena between each testing session.

### Elevated Plus Maze

At P80, animals were tested for general anxiety in the Elevated Plus Maze (EPM). The EPM is raised 55 cm above the ground, containing two closed arms and two open arms and constructed from black Plexiglas®. Rats are videotaped during their session exploring the EPM for 5 minutes. A research analyst, blind to the purpose of the study, recorded how much time the animals spent in the closed arms, open arms, and in the centre. Virkon® was used to clean the arena between each testing session.

### mRNA Analysis

Rats were sacrificed at P80 after all behavioural testing was completed (EPM testing occurred between 9:00–10:30 am and sacrifice occurred between 3:00–5:00 pm). All rats were subjected to isofluorance inhalation, weighed, and decapitated. Using the Zilles atlas^27^ tissue from the PFC, HPC, and NAc was removed, flash frozen on dry ice, and stored at −80 °C for molecular profiling. For molecular analysis of brain tissue, total RNA was extracted from each brain region with the Allprep RNA/DNA Mini Kit according to manufacturer instructions (Qiagen, Germany). The purity and concentration of samples were measured with a NanoDrop 2000 (Thermo Fisher Scientific, USA). Purified RNA (2 μg) was reverse transcribed into cDNA using the oligo(dT)_20_ Superscript III First-Strand Synthesis Supermix Kit (Invritrogen, USA) according to manufacturer protocols.

Genes were selected based on their involvement in dopaminergic and serotonergic pathways along with reward and stress circuitry in the brain. Although this selection of genes is by no means comprehensive, selection was based upon hypothesized roles in the underlying neural circuitry of extinction and reward-based learning. Eight genes were selected: Brain-derived neurotrophic factor, (*Bdnf*), Dopamine receptors 1 and 2, (*Drd1, Drd2*), Glucocorticoid receptor, (*GR*), 5-hydroxytryptamine receptor 1B (*Htr1B*), Ionized calcium-binding adaptor molecule 1, (*Iba1*), Insulin-like growth factor 1, (*Igf1*), Monoamine oxidase A, (*Maoa*), and Mineralocorticoid receptor, (*MR*).

Primers for the qRT-PCR were designed by a research technician in-house, using Primer3 (http://bioinfo.ut.ee/primer3), then purchased from IDT (Coralville, USA). Samples were run in duplicates on a 96-well plate and each target gene was processed. qRT-PCR was performed and analyzed with the Applied Biosystems™ StepOnePlus™ Real-Time PCR System (Thermo Fisher Scientific, USA) with 10 ng of cDNA, 10 μM of the forward and reverse primers for each target gene, and 1X SYBR Green FastMix with Rox (Quanta Biosciences, USA). Two housekeeping genes, CycA and Ywhaz^28^ were used to determine relative target gene expression through the 2^−ΔΔCt^ method as previously described by Pfaffl^29^.

### Serum Biomarker Analysis

Blood was collected at sacrifice (P80) from each rat in serum separator tubes. Samples were clotted at room temperature for 30 minutes then centrifuged at 1000 g for 15 minutes. The separated serum was collected into 300–400 μL samples and stored at −80 °C. ELISA kits were purchased for Testosterone and Corticosterone (Abcam Inc, Canada). ELISAs were performed according to manufacturer’s protocols for each biomarker. All standards, controls and samples were run in duplicate on a 96-well plate and measured using the BioTek Synergy H.T. plate reader and Gen5 2.00.18 software with a path length correction algorithm. Samples were all in normal range of the standard curve.

### Statistical Analysis

All statistical analysis for this experiment was performed with SPSS 22.0 for Mac. Repeated measures ANOVAs were done with Promise Status (kept or broken) and day of training, as factors for performance efficiency. One-way ANOVAs with Promise Status (kept or broken) as a factor were run for all other behavioural tests and the molecular analyses. For all analyses *p* < 0.05 was considered statistically significant.

## Results

### Stage 3 – Efficiency

**Figure 1:**
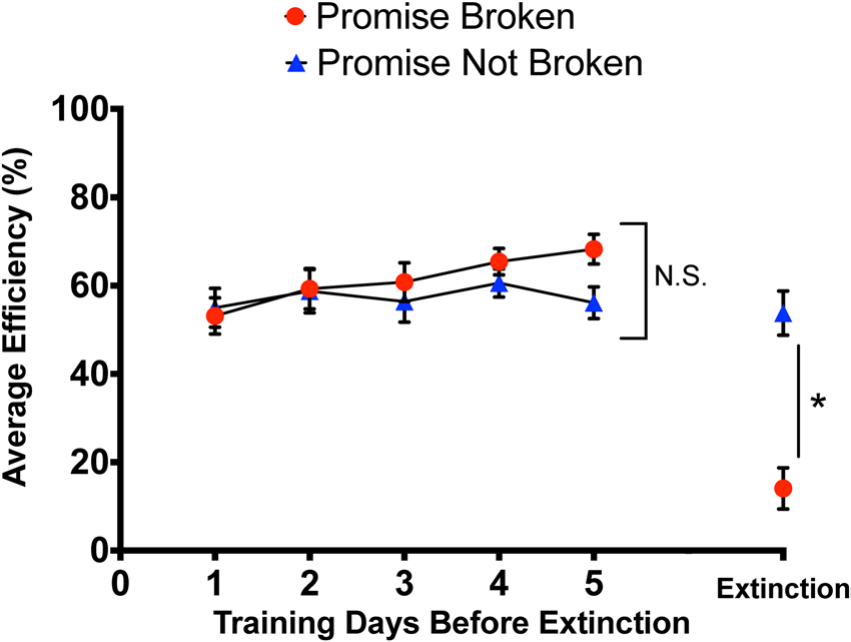
Graphical representation of average efficiency for both groups in the last 5 days of Stage 3 and the average efficiency at extinction. Means ± standard error are displayed where (*) indicates a main effect of group *p* < 0.05 and (N.S.) indicates no significance between groups.

There were no differences in Stage 3 efficiency between the Broken Promise and Kept Promise groups in the 5 days prior to implementation of the extinction protocol (see Fig. 1). The repeated measures ANOVA with days 1–5 and Promise Status as factors failed to demonstrate a significant main effect, *F*(4, 52) = 1.75, *p* = 0.15. There were no significant differences in the total number of trials, the number of successful nose-pokes, or the duration of time spent in time-out *p*’*s* > 0.05.

### Stage 4 –Broken Promise Efficiency

The rats in the Broken Promise group quickly learned that they were no longer being rewarded for completing the task and exhibited significantly reduced efficiencies in the Stage 4 sessions (see Fig. 1). Performance efficiency in Stage 4 for the rats in the Kept Promise group did not differ from their performance in Stage 3. The one-way ANOVA for Stage 4 efficiency exhibited a significant main effect of Promise Status, *F*(1, 15) = 33.50, *p* < 0.01. When comparing the Promise Broken to the Promise Kept group, there were no significant differences in the total number of trials or the duration of time spent in time-out. *p*’s > 05. However, there was a significant difference in the number of successful nose-pokes, whereby animals in the Promise Kept (28.76 ± 5.65) group had significantly more than animals in the Promise Broken group (19.50 ± 3.53), *F*(1, 15) = 4.77, *p* = 0.04.

### Open Field

**Figure 2:**
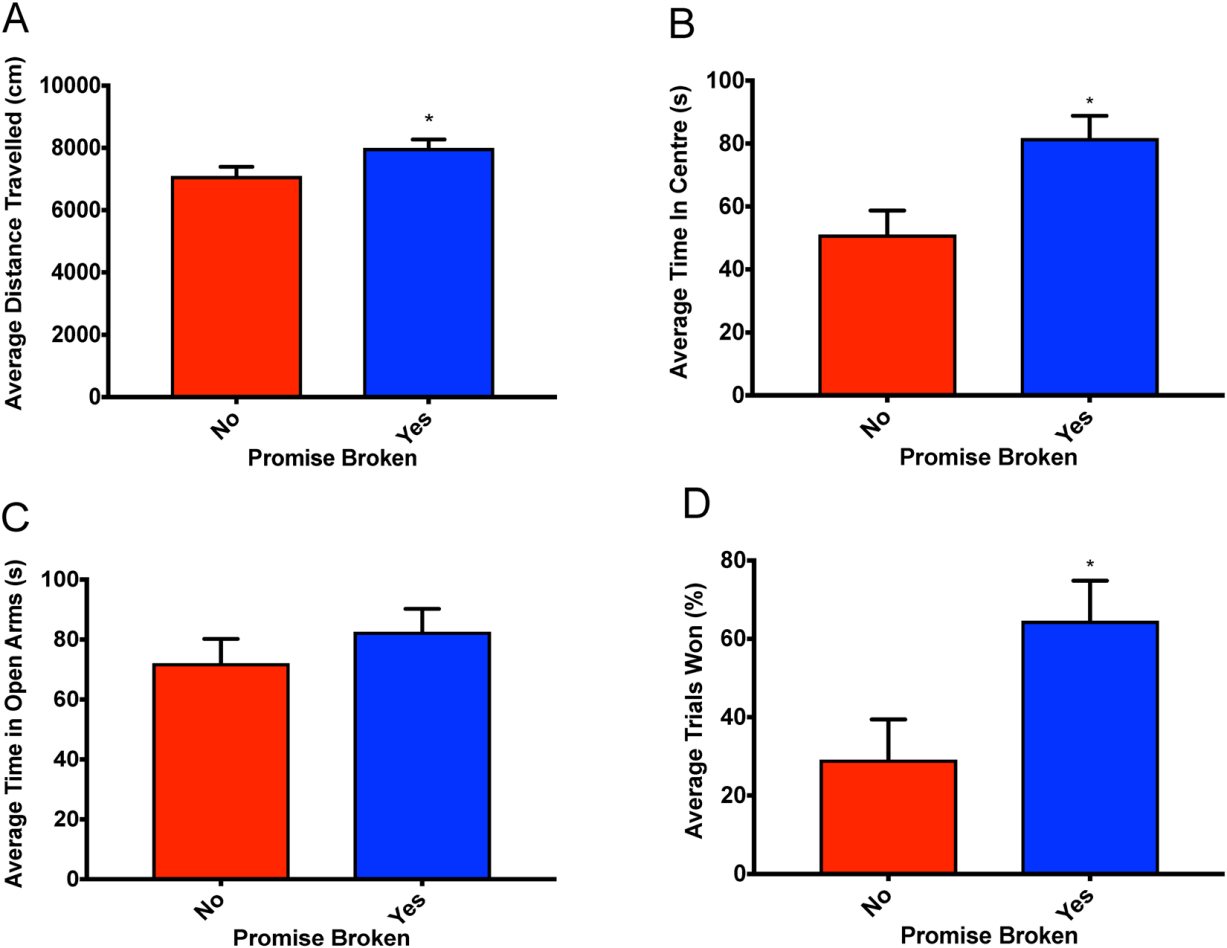
Bar graphs displaying the outcomes from the behavioural test battery for both groups. Means ± standard error are displayed where (*) indicates a main group effect *p* < 0.05. (**A**) Displays the mean distance travelled during the open field test. (**B**) Displays the average time spent in the centre of the arena in the open field test. (**C**) Displays the average time spent in the open arms in the elevated plus maze. (**D**) Displays the average % of trials won in the dominance tube test.

Rats in the Broken Promise group travelled further in the open field and spent significantly more time in the centre of the arena when compared to the rats in the Kept Promise group. The one-way ANOVA for distance travelled demonstrated a significant main effect, *F*(1, 15) = 4.99, *p* < 0.05 (See Fig. 2A), as did the one-way ANOVA for time spent in the centre, *F*(1, 15) = 8.61, *p* < 0.01 (See Fig. 2B).

### Elevated Plus Maze (EPM)

There were no differences in general anxiety levels between Broken Promise and Kept Promise rats as measured with the EPM. The one-way ANOVA for time spent in the open arms of the EPM failed to demonstrate a significant main effect, *F*(1, 15) = 0.89, *p* = 0.36 (See Fig. 2C).

### Dominance Tube

When facing a cage-mate in the opposite group (i.e. Broken Promise vs. Kept Promise), rats in the Broken Promise group were more aggressive and won significantly more trials (65% vs. 29%) than rats in the Kept Promise group. The one-way ANOVA for percentage of trials won in the dominance tube demonstrated a significant main effect, *F*(1, 15) = 41.82, *p* < 0.01 (See Fig. 2D). When facing a cage-mate in the same group (i.e. Broken Promise vs. Broken Promise), win percentage was positively correlated with the rat’s average efficiency during Stage 3. Rats with higher efficiencies were more dominant than rats with lower efficiencies. The Pearson’s correlation between win percentage and Stage 3 efficiency was *r* = 0.504, *p* = 0.02.

### mRNA Expression

**Figure 3:**
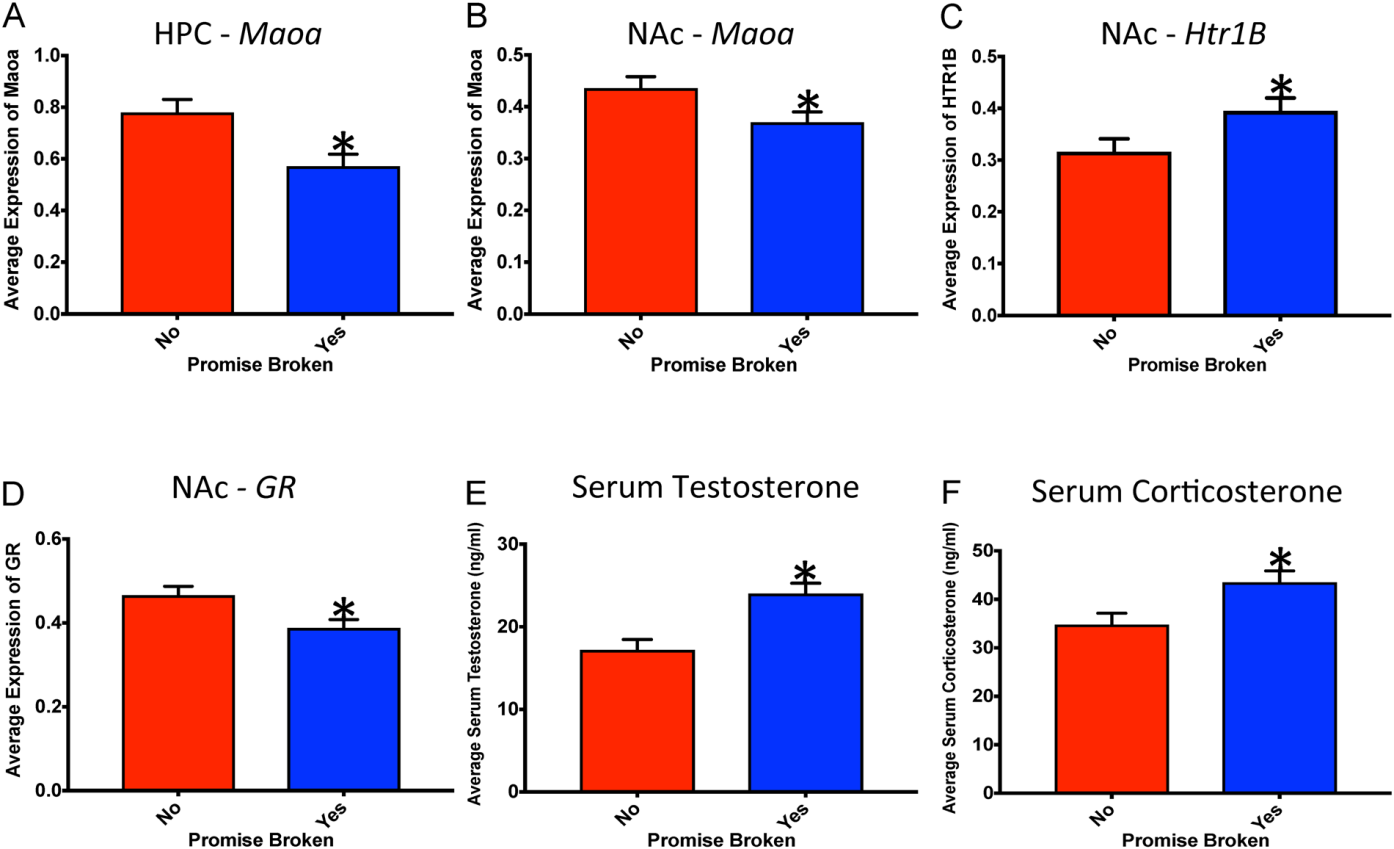
Graphical representation of the average mRNA expression levels in the HPC and NAc and the results from serum ELISAs. Means ± standard error are displayed where (*) indicates a main group effect *p* < 0.05. (**A**) The average mRNA expression of *Maoa* in the HPC. (**B**) The average mRNA expression of *Maoa* in the NAc. (**C**) The average mRNA expression of *Htr1B* in the NAc. (**D**) The average mRNA expression of *GR* in the NAc. (**E**) The mean serum levels of testosterone at the time of sacrifice. (**F**) The mean serum levels of corticosterone at the time of sacrifice.

Gene expression analysis was conducted to investigate neural systems that may be underlying the identified behavioural changes. mRNA expression in the NAc (*GR, Htr1B, Iba1*, and *Maoa*) was influenced to a greater extent than expression in the PFC (only *Drd1*) or HPC (*Htr1B* and *Maoa*). In addition, genes involved in the serotonergic system (*Htr1B* and *Maoa*) were more likely to be significantly altered than the other genes examined. See Table 1 for a summary of mRNA results and See Fig. 3 for graphical representation of the genes with significant changes.

**Table 1:** Summary of statistical results from the one-way ANOVAs for the genes of interest. Bolded text indicates significant main effects.

Gene	Prefrontal Cortex F(*p*)	Hippocampus F(*p*)	Nucleus Accumbens F(*p*)
*Bdnf*	0.05 (0.82)	1.81 (0.20)	1.89 (0.19)
*Drd1*	**11.16** (<**0.01)**	1.31 (0.27)	0.05 (0.95)
*Drd2*	1.38 (0.26)	0.05 (0.83)	0.04 (0.85)
*GR*	1.22 (0.29)	1.19 (0.29)	**7.24 (0.01)**
*Htr1B*	0.91 (0.36)	**14.11 (<0.01)**	**5.07 (0.04)**
*Iba1*	0.44 (0.52)	0.19 (0.67)	**6.61 (0.02)**
*Igf-1*	1.04 (0.33)	0.02 (0.97)	1.12 (0.31)
*Maoa*	0.65 (0.44)	**9.36 (<0.01)**	**4.92 (0.04)**
*MR*	0.19 (0.67)	0.00 (0.97)	1.06 (0.32)

### Serum Biomarkers

Rats in the Broken Promise group had significantly higher levels of serum testosterone than rats in the Promise Kept group when measured at the time of sacrifice. The one-way ANOVA for serum testosterone demonstrated a significant main effect *F*(1, 15) = 14.56, *p* < 0.01 (See Fig. 3A). In addition, rats in the Broken Promise group also had significantly higher levels of serum corticosterone. The one-way ANOVA for serum corticosterone revealed a significant main effect, *F*(1, 15) = 6.89, *p* = 0.02 (See Fig. 3E and F respectively).

## Discussion

In this particular rodent study we found that experiences of broken promises resulted in reduced efficiency in the 3-choice task, increased aggression, and changes in neurological function associated with reward and serotonergic signalling. When promises were broken—the food pellet was no longer provided in response to the correct nose poke—the rats quickly altered their behaviour and stopped performing the task. This finding in and of itself is not novel, because numerous studies have demonstrated that rodents (e.g.^30–32^) and humans alike (for a meta-analysis see^33^) reduce their performance and efforts when the rewarding stimuli has been removed. The innovation in this study stems from the examination of deviant behaviours immediately following this extinction paradigm and loss of reward. In line with predictions of COR theory^8^, rats had elevated levels of corticosterone, engaged in more risky behaviour as demonstrated by increased time in the centre of the open field, and were more aggressive as exhibited by increased win percentages in the dominance tube accompanied by higher levels of testosterone in the aftermath of having experienced a broken promise. As research indicates that aggression and access to agonistic encounters is rewarding and acts as a positive reinforcer^25^, it is possible that rats deprived of reward in the 3-choice serial reaction task were more receptive to the salient reward cues in the dominance tube. Of particular interest was the finding that, win percentage in the dominance tube was positively correlated with rat efficiency in the 3-choice serial reaction task. Although all rats in this group experienced broken promises, rats with higher efficiencies displayed more aggression than rats with lower efficiencies. This is consistent with a series of studies in the field of industrial and organizational psychology, where higher achieving and more committed individuals have more to lose and experience greater stress when promises are broken; it thus seems that “the higher they are, the harder they fall”^34^.

Given that COR theory^8^ postulates a role of stress and the serotonergic system in the deviant behaviours that follow broken promises, we examined mRNA changes in numerous genes associated with the stress response, serotonin, and dopamine, across three distinct brain regions, the PFC, HPC, and the NAc. The most prominent changes in gene expression were associated with serotonin and stress, and were found in the NAc; a brain area particularly involved in motivation, reward, and learning^35^. Consistent with the predictions of COR theory, in the NAc we found alterations to the expression of *GR, Htr1B, Iba1*, and *Maoa*. Changes to expression of *Htr1B* and *Maoa* are consistent with serotonin’s a role in aggression, following physical stress, chronic stress, and social stress (for review see^36^). The modifications in *Htr1B* expression may help explain the deviant behaviours we observed in the dominance tube because studies in mice have demonstrated that *Htr1B* receptors are particularly important for species’ specific aggressive behaviour^37^. The altered expression of *GR* and *Iba1* provide support for the theory that the deviant behaviours that follow the experience of a broken promise result from the perceived stress associated with resource loss, as postulated by COR theory^8^. Glucocorticoid secretion plays a significant role the physiological stress response and in the response to a variety of rewarding stimuli, such as food consumption, receptive sexual partners, and drugs of abuse (for review see^38^). In addition, the role of microglia activation and *Iba1* expression is well established in the stress literature and has been linked to cognitive function and emotional regulation^39^. The combined changes in gene expression identified in the NAc suggest modifications to the reward and social stress response in rats that experienced a broken promise.

Fewer of the genes investigated were altered in the HPC and PFC of rats from the Broken Promise group. However, consistent with the NAc, expression of *Htr1B* and *Maoa* were altered in the HPC as well, suggesting that modulation to the serotoninergic system spanned multiple brain regions. This finding is not surprising as the HPC plays a significant role in social behaviours, the stress response, and reward based learning^40^. Despite findings that both serotonin and dopamine are intimately involved in reward processing, reward value, and reward extinction^22^, the PFC was the only brain region to exhibit changes to the dopaminergic system. mRNA expression of *Drd1* was significantly altered in the PFC of rats that were subjected to broken promises. This identified change in *Drd1* expression could be associated with dopamine’s role in reward or aggression. In mice, dopamine -1 and -2 receptors were found to be involved in the rewarding properties of aggression, with dopamine-1 receptors playing a specific role in the motivation to engage in aggressive behaviours through interactions with the serotoninergic system^25^. This finding would provide further support to the notion that rats in the Broken Promise group were more responsive to the rewarding cues of dominance tube when the rewards in the 3-choice serial reaction task were withdrawn.

In summary, this study offers a major step forward in understanding how and why the experience of a broken promise may lead to the desire to ‘even the score’ by engaging in deviant behaviour. To our knowledge, this study was the first to empirically investigate a widely cited, but never tested, theoretical assumption of COR theory^8^. Furthermore, our initial examination of the mechanisms by which the experience of a broken promise leads to deviant behaviour illustrates a need for further examination of the serotonergic system as it may explain the increase in dominance/aggression and antisocial behavior. To date, only a handful of studies have tried to unpack this association between broken promises and the stress responses, all with very limited self-report questionnaires^5,9,12^. Given our results, we deem it crucial to further investigate the role of one’s serotonergic system in relation to deviant behavior by focusing on more accurate and objective ways of assessing the stress response of humans in the workplace. In conclusion, this study highlights the value and utility of pre-clinical models to examine the theoretical tenants of industrial psychology and offers a promising avenue for future research.

## Data Availability

The data to support the findings of this study are available from the corresponding author upon reasonable request.
